# The prognostic value of pre-operative coronary evaluation in kidney transplanted patients

**DOI:** 10.3389/fcvm.2022.974158

**Published:** 2022-08-05

**Authors:** Tali Steinmetz, Leor Perl, Benaya Rozen Zvi, Mohamad Atamna, Ran Kornowski, Arthur Shiyovich, Ashraf Hamdan, Eviatar Nesher, Ruth Rahamimov, Tuvia Ben Gal, Keren Skalsky

**Affiliations:** ^1^Department of Nephrology, Rabin Medical Center, Petach-Tikva, Israel; ^2^Affiliated to the Faculty of Medicine, Tel Aviv University, Tel Aviv, Israel; ^3^Department of Cardiology, Rabin Medical Center, Petach-Tikva, Israel; ^4^Department of Transplantation, Rabin Medical Center, Petach-Tikva, Israel

**Keywords:** kidney transplant, pre-surgical cardiac evaluation, nuclear SPECT test, chronic kidney disease, coronary artery disease

## Abstract

**Aims:**

Non-invasive coronary assessment using single-photon emission computerized tomography (SPECT) testing for potential cardiac ischemia is an essential part of the evaluation of kidney transplant candidates. We aimed to examine the prognostic value of preoperative SPECT test results in kidney transplanted patients.

**Methods and results:**

We retrospectively analyzed the pre-surgical nuclear SPECT test results in a registry of kidney transplanted patients. Follow-up at 1 month and 1 year recorded major adverse cardiac events (MACE) including non-fatal myocardial infarction, all-cause mortality and hospitalization due to cardiovascular disease following the renal transplantation. Of 577 patients available for analysis, 408 (70.9%) patients underwent nuclear SPECT test pre-transplant and 83 (20.3%) had abnormal results with either evidence of ischemia or infarct. A significantly higher incidence of post-operative MACE at 1 month was evident among patients with abnormal SPECT test compared to patients with no evidence of ischemia (10.8 vs. 4.3% respectively; *P* = 0.019). Differences were mostly derived from significantly increased rates of myocardial infarction events (8.4 vs. 1.8%; P = 0.002). Yet, MACE rate was not statistically different at 1 year (20.5 vs. 13.1%; *P* = 0.88). Importantly, the prognostic impact of an abnormal SPECT was significantly attenuated for all outcomes following multivariable adjusting for conventional cardiovascular risk factors and coronary revascularization.

**Conclusion:**

Pre-surgical cardiac risk assessment of kidney transplant candidates with nuclear SPECT test was found to be predictive of post-operative MACE, yet apparently, its prognostic value was significantly attenuated when adjusted for cardiac risk factors.

## Introduction

An important part of the pre-surgical evaluation of kidney transplant candidates (KTCs) includes assessment for a coronary disease, as chronic kidney failure patients are at excessive risk for coronary artery disease (CAD) (1–3). Indeed, KTCs with ESRD who had undergone cardiac assessment showed an increased prevalence of cardiovascular morbidity and mortality both pre- and post- kidney transplantation (4, 5).

Thus, although kidney transplantation is considered intermediate risk surgery (the risk for 30-day mortality or non-fatal MI after kidney transplantation is only 1–5%) (6), in most KTCs, the CVD risk is further increased, due to burden of renal-cardiac-metabolic co-morbidities (e.g., diabetes mellitus, hypertension, dyslipidemia and peripheral vascular disease) often present. Previous cardiovascular events and longer dialysis treatment further augment the hazard. In addition, the peri-transplant period is challenging for both the heart and the transplanted kidney due to rapid hemodynamic changes (7), fluid overload, anemia (8), and electrolyte abnormalities (9). Perioperative myocardial ischemia may require invasive coronary angiogram, contrast exposure and antiplatelet therapy, which can pose a risk to the renal allograft (10–12).

Following recommended guidelines of the preferred diagnostic method for perioperative cardiovascular evaluation (13–16); over the past 15 years, most KTCs at our Medical Center have routinely undergone non-invasive coronary assessment using nuclear single photon emission computed tomography (SPECT) imaging with either Bruce protocol or vasodilator stress test, according to their functional status. Additionally, patients over 50 years old, with chronic coronary artery disease or major cardiovascular risk also had a cardiologist evaluation and accordingly coronary angiography was performed.

Nonetheless, despite efforts at examination and validation throughout the past 2 decades, current evidence on the association between SPECT test results in KTCs and their clinical cardiac outcomes following transplantation is still conflicting (6, 17–21). The current study is thus yet another attempt to solve this conundrum of a valid test methodology and technology (e.g., non-invasive stress tests, as SPECT test) that simply does not deliver high enough sensitivity and specificity in renal failure patients, candidates for kidney transplant. Therefore, in the current study, our objectives were:

1. to examine the prognostic value of preoperative cardiac evaluation for myocardial ischemia among kidney transplanted patients and

2. to assess the correlation between the SPECT results and prognostic measures following kidney transplantation.

## Methods

### Study design

The present study was a retrospective analysis of prospective data from a cohort of patients treated at one of the two institutions (Beilenson and Hasharon hospitals) at Rabin Medical Center, who underwent kidney transplantation between the years 2016 and 2019. Patients included in the analysis were all 18 years old or above. Exclusion criteria included: concomitant heart or liver transplantation.

The data collected from the registry for each patient included demographics (age and gender) and medical history: comorbidities [diabetes, hypertension, and lipid profile], smoking, chronic obstructive pulmonary disease (COPD), peripheral vascular disease (PVD), cerebrovascular accidents (CVA)], the presence of arteriovenous fistula (AVF), duration of dialysis and allograft source (living vs. cadaver).

Pre-transplant cardiac investigation data collected included: nuclear SPECT scan protocol (thallium-201/technetium 99 m sestamibi, bruce protocol/dipyridamole), and cardiac results (i.e., reversible ischemia, infarcts and transient ischemic dilation); coronary angiographic findings, basic echocardiographic data (estimated ejection fraction, valvular regurgitation and stenosis and estimated systolic pulmonary pressure) and clinical events. Mortality outcome was retrieved through the hospital administration system (ATD) which is updated by the Israel's Ministry of Health's registration.

For the analyses patients were categorized according to the preoperative evaluation with nuclear SPECT test results. A SPECT scan with reversible ischemia, perfusion defects or transient ischemic dilatation was considered positive. The study protocol was approved by the local Institutional Review Board of our medical center.

### Study endpoints

Major advance cardiac events (MACE) at 1 month and 1-year post-transplantation was considered the primary endpoint. Clinical outcomes: MACE, comprising non-fatal myocardial infarction (MI), all-cause mortality and hospitalization due to cardiovascular disease following renal transplantation were retrospectively collected from the institutional database, or when indicated, records from other hospitals were acquired to verify the events in the follow-up period. All events were further confirmed by two researchers (KS, TS). Survival status at follow-up was assessed by review of ATD registries up to 3 years. MI was defined according to the forth universal definition of MI type 1 or type 2 (19).

### Statistical analysis

Continuous data are summarized as mean and standard deviation (SD) or median and interquartile range (IQR) and were compared using Student t tests or analyses of variance. Categorical variables are presented as frequency and were compared by chi-square or Fisher's exact tests. The normality of variable distributions was assessed using the Kolmogorov–Smirnov test. Time-to-event curves were constructed using the Kaplan–Meier method and compared using log-rank test. Cox regression analyses were performed to identify independent predictors of the primary end point. Covariates for the Cox model were chosen according to their known association with myocardial ischemia and clinical outcomes, and included age, sex, diabetes mellitus, the presence of an AV-fistula, duration of dialysis, known ischemic heart disease, percutaneous coronary intervention (PCI) and the presence of ischemia as evident in the SPECT testing. Effect sizes are presented as hazard ratios and 95% confidence intervals. All statistical analyses were performed with IBM SPSS statistics V.27 software. A *P*-value of <0.05 was considered statistically significant.

## Results

A total of 577 patients were available for analysis. Of them, 408 had nuclear SPECT test results, as part of the pre-transplant cardiac assessment. As presented in Table 1 compared to the non-tested group, patients who had a nuclear SPECT test were significantly older, had higher prevalence of cardio-metabolic comorbidities and longer duration of dialysis. While the non-tested group of younger patients had significantly higher rates receiving living donor transplant. Correspondingly, the rates of patients who required a pre-transplant coronary angiography were significantly higher 13.245.3 vs.13.2% in the nuclear SPECT test group (*p* < 0.001). Importantly, the rate of MACE was significantly higher 13.0 vs. 1.8% in the SPECT test group (*p* < 0.001).

**Table 1:** Patients demographic and Baseline pre-transplant clinical characteristics according to cardiac assessment with/without SPECT scan.

**Variable**	**Scan *n* = 408**	**No Scan *n* = 167**	***P*-value**
**Age (mean, years)**	**56.75 ± 12.28**	**36.78 ± 13.41**	**<0.01**
Male gender (%)	69.1	64.1	0.24
Dialysis (%)	88.7	72.5	<0.01
Duration of dialysis (mean, months)	50.68 ± 43.65	30.24 ± 37.10	<0.01
AV fistula (%)	55.6	31.7	<0.01
Living donor (%)	56.1	80.8	<0.01
Diabetes (%)	69.1	21.6	<0.01
Hypertension (%)	91.8	75.3	<0.01
BMI (kg/m^2^)	28.53 ± 26.71	23.49 ± 4.33	0.02
Known IHD	29.7	1.8	<0.01
PVD (%)	7.7	1.3	0.01
CVA (%)	7.9	1.8	0.01
Dyslipidemia (%)	61.6	24.0	<0.01
LDL cholesterol (mean, mg/dl)	87.07 ± 36.52	88.53 ± 36.93	0.66
HDL cholesterol (mean, mg/dl)	43.64 ± 14.85	45.47 ± 15.65	0.19
Current smoker (%)	44.4	34.7	0.15
Pre-transplant AF (%)	6.7	0.6	<0.01

*SPECT, single-photon emission computerized tomography; AV fistula, arteriovenous fistula; BMI, body mass index; PVD, peripheral vascular disease; CVA, cerebrovascular accidents; LDL cholesterol, low-density lipoprotein; HDL cholesterol, high-density lipoprotein; AF, atrial fibrillation*.

Of the 408 patients who underwent nuclear SPECT test, 83 (20.3%) had a positive SPECT result, with evidence for ischemia of any degree, fixed perfusion defects or transient ischemic dilatation. Technetium was used as the radioactive substance more frequently among patients with negative SPECT results, compared to patients in the positive SPECT tests, and Bruce protocol was more frequently used in the latter group (Table 2). Comparison of groups according to SPECT scan results as depicted in Table 3, patients with abnormal SPECT results tend to be older males, with higher prevalence of cardio-metabolic comorbidities. The average time interval between the SPECT scan to transplant was 8.4 ± 7.1 months in the positive scan group and 9.6 ± 8.8 months in the negative scan group (*P* = 0.28). Seventy one out of 83 (85.5%) patients in the abnormal SPECT test group underwent an invasive coronary angiography procedure prior to transplant, compared to 109 out of 316 (34.5%) in the normal SPECT group (*p*< 0.001). Rates of PCI performed were 33.7 and 7.1%, respectively (*p*< 0.001).

**Table 2:** SPECT scan characteristics of patient groups according to SPECT scan results.

**Variable**	**Positive SPECT scan *n* = 83**	**Negative SPECT scan *n* = 316**	***P*-value**
Technetium (%)	65.2	48.6	<0.01
Bruce protocol (%)	27.4	38.0	0.05
Pulse at rest (mean, beats per minute)	74.1 ± 12.4	76.6 ± 12.5	0.07
Pulse at stress (mean, beats per minute)	99.6 ± 27.0	111.6 ± 33.1	<0.01
Systolic pressure at rest (mean, mmHg)	140.9 ± 21.3	137.9 ± 24.5	0.17
Systolic pressure at stress (mean, mmHg)	141.4 ± 30.0	145.5 ± 30.0	0.15
Metabolic equivalents (METS; mean)	7.0 ± 3.5	8.3 ± 3.7	0.11

**Table 3:** Baseline characteristics of patient groups according to SPECT scan results.

**Variable**	**Positive SPECT Scan *n* = 83**	**Negative SPECT Scan *n* = 316**	***P*-value**
Age (mean, years)	61.52+-10.08	49.02+-15.73	<0.01
Male gender (%)	80.7	66.2	<0.01
Dialysis (%)	86.6	83.6	0.51
Duration of dialysis (mean, months)	47.15 ± 35.82	44.38 ± 43.61	0.61
AV fistula (%)	59.0	46.4	0.03
Living donor (%)	57.8	61.9	0.48
Diabetes (%)	96.4	48.8	<0.01
Hypertension (%)	96.4	84.8	0.01
BMI (kg/m^2^)	27.98 ± 4.5	26.8 ± 23.92	0.65
Known IHD	61.14	15.1	<0.01
PVD (%)	10.7	5.1	0.06
CVA (%)	8.5	5.6	0.31
Dyslipidemia (%)	80.7	45.3	<0.01
LDL cholesterol (mean, mg/dl)	90.99 ± 37.19	86.72 ± 35.49	0.32
HDL cholesterol (mean, mg/dl)	42.28 ± 3.99	44.47 ± 15.30	0.22
Current smoker (%)	44.6	41.5	0.72
Pre-transplant AF (%)	8.5	4.3	0.10

*SPECT, single-photon emission computerized tomography; AV fistula, arteriovenous fistula; BMI, body mass index; PVD, peripheral vascular disease; CVA, cerebrovascular accidents; LDL cholesterol, low-density lipoprotein; HDL cholesterol, high-density lipoprotein; AF,- atrial fibrillation*.

**Figure 1:**
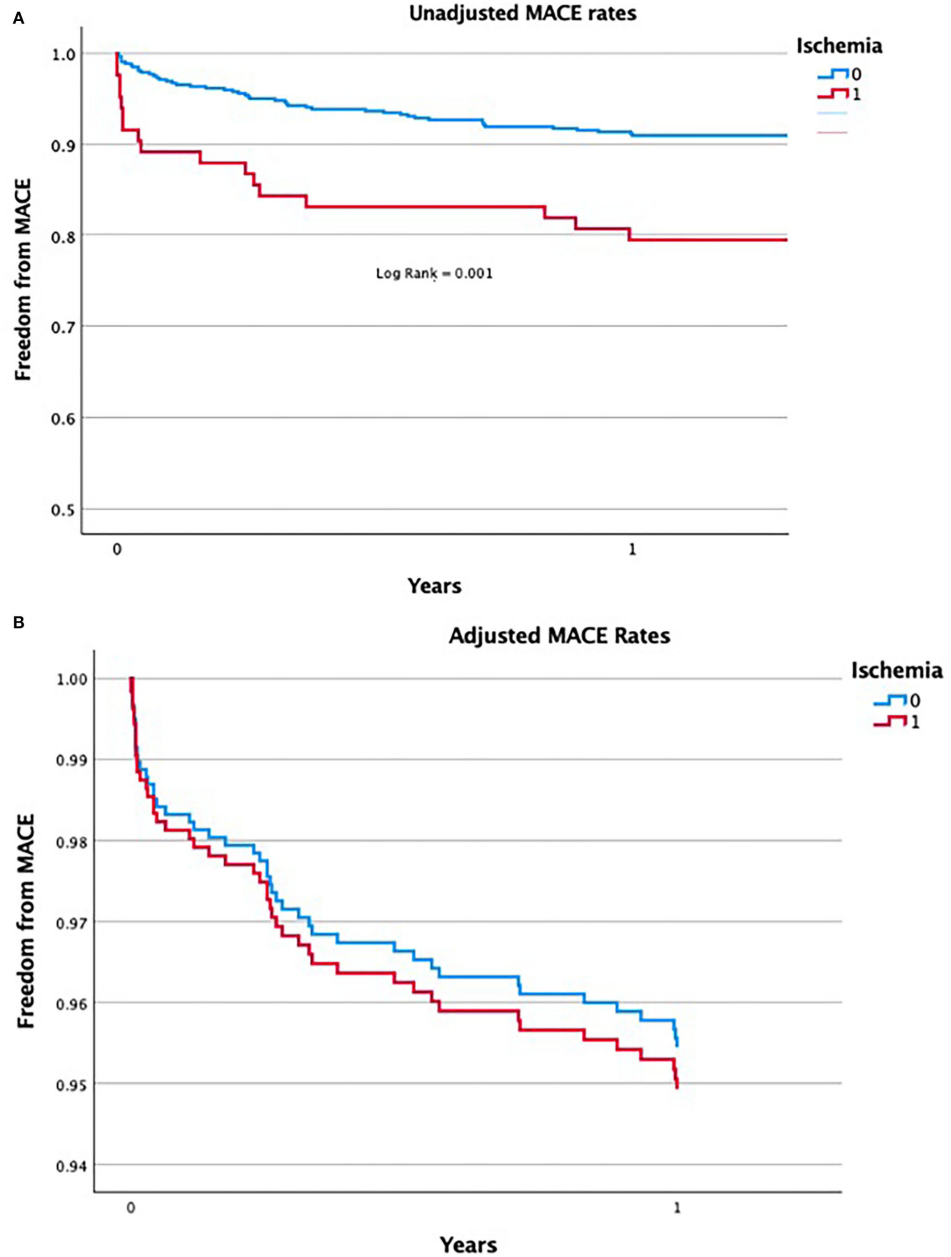
**(A)** Unadjusted rates of MACE in patients with abnormal SPECT test results compared to patients with normal SPECT scan. **(B)** Adjusted rates of MACE in patients with abnormal SPECT test results compared to patients with normal SPECT scan.

The incidence of MACE at 1 month was significantly higher among patients with abnormal SPECT test compared to patients with no evidence of ischemia (10.8 vs. 4.3% respectively; *P* = 0.019). Differences were mostly derived by increased rates of post-transplant myocardial infarction (8.4 vs. 1.8%; *P* = 0.002). Yet the MACE rate was no longer statistically different at 1 year following kidney transplantation (20.5 vs. 13.1%; *P* = 0.88). Moreover, as depicted in Figures 1A,B when adjusting the results according to conventional cardiovascular risk factors and according to the coronary intervention (as specified above), the prognostic impact of an abnormal SPECT had been attenuated for all outcomes.

Notwithstanding, risk factors were found predictive for MACE at 1 year including: patient age (OR 1.06 for each additional year 95% CI 1.03–1.09; *P*< 0.001), male gender (OR 1.75 95% CI 1.18–1.85; *P* = 0.016), prior history of ischemic heart disease (OR 2.44 95% CI 1.3–4.6; *P* = 0.006), duration of dialysis (OR 1.008 for each additional month under dialysis 95% CI 1.002–1.015; *P*< 0.01), and the presence of AV fistula (OR 2.4 95%CI 1.12–5.17; *P* = 0.02). PCI and diabetes were not among the predictors of 1 year MACE (Table 4).

**Table 4:** Logistic regression analysis for MACE.

	**Odds ratio**	**95% confidence interval**	***P*-value**
Age	1.06	1.03–1.09	<0.001
Male gender	1.75	1.18–1.85	0.016
Known ischemic heart disease	2.44	1.3–4.6	0.006
Duration of dialysis	1.008	1.002–1.015	<0.001
AV fistula	2.4	1.12–5.17	0.02
Diabetes	1.08	0.81–1.45	0.59
PCI	0.55	0.51–1.7	0.32

*MACE, major adverse cardiac events; AV fistula, arteriovenous fistula; PCI, percutaneous coronary intervention*.

## Discussion

Our study demonstrate the prediction value of pre-surgical cardiac evaluation of kidney transplant candidates with nuclear SPECT test. We have found the test to be predictive of post-operative MACE, although its prognostic value was attenuated significantly when adjusted for risk factors. The study expands upon previous observations with an updated analysis of a real-world large cohort of kidney transplant patients and their post-surgical cardiovascular outcomes. The results may further challenge clinicians evaluating KTCs for transplant, as positive SPECT test was not found to have a significant prognostic impact after adjustment for risk factors and for pre-transplant coronary revascularization. We suggest to rely on cardiovascular risk assessment and subjective clinical impression, with a high index of suspicion aimed toward male candidates with advanced age, long duration of dialysis and known IHD, as these were found to be independent predictors of MACE. A positive SPECT scan may represent all of these predictors and thus can support a clinical decision to proceed to coronary angiography.

In the first analysis we found that patients who had undergone nuclear SPECT test, as part of their pre-surgical evaluation, tended to be older with significantly higher rates of cardio-metabolic comorbidities compared to those with no prior cardiac functional test. The rates of 1 year MACE were higher in this group, as expected, according to their augmented risk factors. In the second analysis, we first compared rates of MACE among patients with abnormal SPECT tests vs. those with normal SPECT imaging results. Analysis of the raw data implied that an abnormal SPECT scan has a prognostic value with increased risk of MACE at 1-month post-surgery. However, following adjustment for confounding risk factors, the positive SPECT test was no longer predictive of MACE at 1 month or even at 1 year.

Previous publications suggested that positive results on non-invasive stress testing in patients assessed for kidney transplantation is predictive of cardiovascular mortality (6, 17). However, the evidence relies on data published in a limited number of studies using different non-invasive tests (18). A prognostic assessment of the SPECT scan was performed in two studies a decade ago with conflicting results. The first, a small cohort published in 2002, examined 61 KTCs, found a positive SPECT scan was predictive of postoperative MI, however, their results were not adjusted for cofounders (20). The second, a prospective cohort of 126 KTCs from 2003 compared the prognostic significance of coronary angiography to SPECT scan and dubotamine stress echocardiography. Critical coronary lesions seen on coronary angiography were found to be the sole predictor of cardiac events (21). A more recent publication compared the prognostic value of coronary computed tomography angiography (CTA), coronary artery calcium score, SPECT scan and invasive coronary angiography and in contrast to our results, found that coronary CTA and invasive coronary angiography were predictive of MACE whereas SPECT scan was not a determinant in the risk of MACE (22). A large prospective cohort of 564 patients in Finland undergoing kidney transplant, found, compared to a normal finding on SPECT, pre-surgical ischemia detected on SPECT scan predicted a 2-fold risk of all-cause and cardiovascular mortality post-transplant (23).

Results are not unexpected as the sensitivity and specificity of non-invasive stress tests, including SPECT test in renal failure patients are relatively low. Positive SPECT results not reflected on coronary angiography may be a consequence of microvascular disease and/or endothelial dysfunction syndrome. On the other hand, false negative results might have a few explanations: the presence of multi-vessel disease resulting in balanced\global ischemia; the presence of collateral vessels that produce the compensatory appearance of more homogeneous perfusion; and higher resting levels of adenosine, resulting in higher resting coronary flow and reduced responsiveness to dipyridamole, causing inadequate functional stress myocardial response (24).

Whether coronary angiography and/or revascularization during the pre-transplant evaluation could prevent future cardiovascular events is debatable. Two randomized control trials previously addressed this issue: a small trial published in 1992 and a much more recent, *post-hoc* analysis of the ISCHEMIA-CKD trial published in 2020. In the first study, 26 kidney transplant candidates with diabetes and obstructive CAD were randomly assigned to medical therapy vs. revascularization. A combined cardiovascular endpoint occurred in 10 of 13 medically managed patients, compared to 2 of 13 re-vascularized patients, within 8.4 months (25). Nevertheless, the timing of transplant in this small study was not reported and results post-transplant are unknown. The second publication, a *post-hoc* analysis of the randomized control ISCHEMIA-CKD trial, examined intervention vs. conservative management in 194 participants listed for transplant compared to 583 patients not listed. Pre-emptive coronary revascularization in patients on the waiting list was not associated with improved survival, although only a minority of patients had kidney transplant and there was no reported data of outcomes post-surgery (26).

The study has a few limitations. Due to the observational design, there is a clear selection bias of patients to undergo nuclear stress test and coronary angiography. The characteristics of patients in the different groups may vary and possibly could not be completely corrected using multivariate analysis. Moreover, revascularizations were performed more frequently among patients with positive SPECT scans, which might have an impact on the study results. Nevertheless, this is an updated real-world study that assessed the occurrence of MACE and the predictive value of abnormal nuclear stress test in the population of patients undergoing kidney transplant.

Overall, decisions regarding the pre-transplant cardiac evaluation of KTCs should be tailored made with careful consideration of the patient‘s complains, medical history, laboratory tests, images, renal function and prognosis. Treatment considerations in patients on the waiting list should include the potential future damage to the transplanted kidney with the need of performing coronary revascularization post-surgery. The “heart team” forum should consult with the nephrologists how to optimize the medical care for the heart and kidneys together.

## Conclusion

Pre-surgical cardiac evaluation of kidney transplant candidates, with nuclear SPECT test was found to be predictive of post-operative MACE, although its prognostic value was attenuated significantly, when adjusted for risk factors. A personalized clinical assessment, in which the SPECT scan results would be a decision supporting tool rather than a single and exclusive criteria, should be considered to guide treatment.

## Data availability statement

The original contributions presented in the study are included in the article/supplementary material, further inquiries can be directed to the corresponding author/s.

## Ethics statement

The studies involving human participants were reviewed and approved by 0151-19-RMC. Written informed consent for participation was not required for this study in accordance with the national legislation and the institutional requirements.

## Author contributions

TS: writing—original draft. LP: methodology and software. BZ: writing—review and editing, methodology, and software. MA: data curation. RK and AH: investigation and supervision. AS: writing—review and editing. EN, RR, and TG: visualization. KS: conceptualization and writing—original draft. All authors have read and approved the manuscript.

## Conflict of interest

The authors declare that the research was conducted in the absence of any commercial or financial relationships that could be construed as a potential conflict of interest.

## Publisher's note

All claims expressed in this article are solely those of the authors and do not necessarily represent those of their affiliated organizations, or those of the publisher, the editors and the reviewers. Any product that may be evaluated in this article, or claim that may be made by its manufacturer, is not guaranteed or endorsed by the publisher.
